# Application of tranexamic acid during debridement for proximal-to-mid humeral chronic osteomyelitis: evaluation of blood management and infection-control safety

**DOI:** 10.3389/fsurg.2026.1933516

**Published:** 2026-09-24

**Authors:** Xingyu Chen, Chuntao Zhao, Wei Zhang, Zhongzheng Yu, Xijiao Zhang, Hu Zhang, Hangyu Li, Guohao Liu, Xiaoxiao Song, Jian Shi, Xiaoyong Yang

**Affiliations:** 1 Department of Orthopaedics, The 920th Hospital of Joint Logistics Support Force of Chinese People’s Liberation Army, Kunming, Yunnan, China; 2 Yunnan Provincial Key Laboratory of Microsurgical Medical Technology, The 920th Hospital of Joint Logistics Support Force of Chinese People's Liberation Army, Kunming, Yunnan, China; 3 Department of Statistics, Kunming Medical University, Kunming, Yunnan, China

**Keywords:** blood management, chronic osteomyelitis, debridement, frozen section, humerus, infection control, tranexamic acid, venous thromboembolism

## Abstract

**Background:**

Adequate exposure and thorough removal of infected tissue are essential during debridement for chronic osteomyelitis. In proximal-to-mid humeral chronic osteomyelitis, complex anatomy, abundant musculature, and limited tourniquet feasibility may cause diffuse bleeding, impaired visualization, and prolonged surgery. Although tranexamic acid (TXA) is widely used for orthopaedic blood management, its infection-control safety in infected bone debridement remains unclear. We evaluated preoperative intravenous TXA in this setting.

**Methods:**

This single-center retrospective cohort study included patients undergoing debridement for chronic osteomyelitis of the proximal or mid-humerus between January 2020 and March 2025. Patients were grouped by preoperative intravenous TXA use; the TXA group received 1.0 g approximately 30 min before surgery. Primary outcomes were second-stage frozen-section neutrophil count, unplanned repeat debridement, infection recurrence, and venous thromboembolic events. Secondary outcomes were operative time, estimated intraoperative blood loss, postoperative drainage volume, recorded perioperative blood loss, and transfusion.

**Results:**

Eighty-two patients were included: 47 received TXA and 35 did not. Baseline characteristics were generally comparable. Operative time (139.2 ± 13.4 vs. 148.7 ± 11.8 min, *P* = 0.001), estimated intraoperative blood loss (289 ± 35 vs. 346 ± 49 mL, *P* < 0.001), postoperative drainage volume (160 ± 26 vs. 179 ± 23 mL, *P* < 0.001), and recorded perioperative blood loss (449 ± 47 vs. 525 ± 53 mL, *P* < 0.001) were lower in the TXA group. Transfusion rates did not differ significantly (8.5% vs. 14.3%, *P* = 0.486). Second-stage frozen-section neutrophil counts were similar (2.0 [1.0, 2.0] vs. 2.0 [1.0, 3.0], *P* = 0.106). Unplanned repeat debridement before the planned second-stage procedure occurred in 8.5% and 8.6% of patients (*P* = 1.000), respectively, and infection recurrence during the observation period occurred in 8.5% and 11.4% (*P* = 0.718), respectively. All recurrences occurred before the planned second-stage procedure; none occurred during at least 12 months of subsequent post-second-stage follow-up.

**Conclusions:**

In selected patients without TXA contraindications, preoperative intravenous TXA was associated with lower perioperative blood-loss measures and shorter operative time, with no statistically detectable increase in infection-control adverse signals. No clinically diagnosed venous thromboembolic events were documented during follow-up.

## Introduction

Chronic osteomyelitis remains one of the most challenging conditions in orthopaedic infection, owing to its prolonged disease course, occult infectious foci, high risk of recurrence, and frequent need for staged surgical treatment (1–4). For chronic osteomyelitis involving the proximal-to-mid humerus, thorough debridement is a prerequisite for infection control and subsequent reconstruction (5–7). However, this anatomical region is characterized by complex neurovascular structures, abundant surrounding musculature, and limited feasibility of achieving a stable bloodless field with a tourniquet. Therefore, diffuse bleeding during first-stage debridement may not only increase perioperative blood loss and transfusion requirements, but may also impair visualization of the operative field and recognition of infected tissue boundaries, reduce the technical controllability of debridement, and prolong operative time (3, 6).

Tranexamic acid (TXA) is an antifibrinolytic agent that reduces bleeding by inhibiting plasminogen activation and fibrin degradation (8). It has been widely used in total joint arthroplasty, orthopaedic trauma, spine surgery, and other non-infective or relatively aseptic surgical settings, where its blood-sparing effect and ability to reduce transfusion requirements have been well established (9–13). Nevertheless, direct evidence supporting TXA use in infective orthopaedic procedures, particularly debridement for chronic osteomyelitis, remains limited. Unlike elective aseptic surgery, infective debridement is performed in a surgical environment that may contain active infectious foci, necrotic tissue, infected granulation tissue, postoperative hematoma risk, and potentially residual bacteria (1, 5, 6). By stabilizing fibrin clots, TXA may theoretically reduce hematoma formation and wound oozing; however, whether a more stable clot- and fibrin-rich microenvironment could impair residual bacterial clearance, antibiotic penetration, or infection control remains uncertain (8).

Recent studies in arthroplasty have suggested that TXA does not increase postoperative infection risk and may even be associated with a lower risk of periprosthetic joint infection (PJI) (14, 15). However, this evidence is mainly derived from primary total hip and knee arthroplasty, where the baseline infection burden, surgical environment, host condition, and infection-control objectives differ substantially from those in chronic osteomyelitis debridement. Therefore, conclusions from arthroplasty should not be directly extrapolated to infected bone surgery. In the context of humeral chronic osteomyelitis debridement, the central question is not merely whether TXA has a conventional hemostatic effect, but whether TXA is associated with unfavorable infection-control signals, such as persistent local inflammation, higher second-stage frozen-section neutrophil counts, unplanned repeat debridement, or infection recurrence (16, 17).

Based on this rationale, the present retrospective cohort study evaluated patients who underwent debridement for chronic osteomyelitis of the proximal-to-mid humerus. We compared infection-control-related outcomes and perioperative safety between patients who received preoperative intravenous TXA and those who did not. The primary focus was to determine whether TXA use was associated with unfavorable infection-control signals, including higher second-stage frozen-section neutrophil counts, unplanned repeat debridement, and infection recurrence during the observation period; venous thromboembolism was assessed separately as a thromboembolic safety outcome. Secondary exploratory outcomes included operative time, estimated intraoperative blood loss, postoperative drainage volume, recorded perioperative blood loss, and transfusion requirement. This study aims to provide preliminary clinical evidence regarding the infection-control safety and perioperative value of TXA in infective debridement for humeral chronic osteomyelitis.

## Materials and methods

### Study design and patients

This was a single-center retrospective cohort study. Potentially eligible patients were identified from the hospital electronic medical record system. We retrospectively reviewed patients who underwent surgical treatment for chronic osteomyelitis of the proximal and mid-humerus at our institution between January 2020 and March 2025. Patients were classified into the TXA and non-TXA groups according to whether intravenous tranexamic acid (TXA) was administered before first-stage debridement. TXA exposure was ascertained from perioperative medication orders and administration records. During the study period, there was no standardized institutional protocol or predefined study-based allocation rule governing the use of TXA during debridement for chronic osteomyelitis. TXA exposure was determined retrospectively from perioperative medication orders and administration records. The decision to prescribe TXA was made by the treating ward physician as part of routine perioperative care and varied according to individual prescribing practice. Because the specific rationale for TXA administration was not systematically documented in the medical records, the reason for treatment selection could not be reconstructed for every individual patient. This study was approved by the institutional ethics committee of our hospital (No. 2024-072-01).

The inclusion criteria were as follows: (1) age ≥18 years; (2) diagnosis of chronic osteomyelitis involving the proximal or mid-humerus; (3) treatment with first-stage infective debridement and local antibiotic cement-based dead-space management; (4) complete perioperative data, infection-control-related outcomes, and follow-up data; and (5) follow-up of at least 12 months after the planned second-stage procedure. The exclusion criteria were as follows: (1) lesions involving the distal humerus; (2) acute hematogenous osteomyelitis, malignant bone or soft-tissue tumors, osteoarticular tuberculosis, or other specific infections; (3) incomplete clinical data or follow-up of less than 12 months after the planned second-stage procedure; and (4) active thrombosis, a history of venous thromboembolism, long-term anticoagulant therapy, obvious coagulation abnormalities, severe hepatic or renal dysfunction, epilepsy, or other contraindications to TXA.

Bone infection was diagnosed using clinical, imaging, microbiological, histopathological, and intraoperative evidence. For fracture-related cases, infection was assessed according to the international fracture-related infection (FRI) consensus definition (5); for non-fracture-related peripheral bone infections, diagnostic assessment was based on the EANM/EBJIS/ESR consensus framework (18). Cases were classified as chronic osteomyelitis based on a persistent or recurrent clinical course together with clinical, radiological, and/or intraoperative features characteristic of chronic bone infection, including sinus tract formation, sequestrum, sclerosis, or necrotic bone (2). Laboratory inflammatory markers were considered supportive rather than definitive diagnostic evidence. Preoperative radiographs, CT, and/or MRI were used to characterize the extent of osseous involvement, sequestrum formation, intramedullary extension, bone defects, and surrounding soft-tissue conditions.

### TXA exposure

Patients in the TXA group received a single dose of 1.0 g TXA diluted in 100 mL of normal saline by intravenous infusion. For patients in whom TXA was prescribed, the infusion was initiated approximately 30 min before skin incision and completed within approximately 15 min to allow the intravenous dose to be fully administered before the start of surgery. This timing reflected routine perioperative practice at our institution during the study period and was not selected according to a study-specific pharmacokinetic protocol. No additional intraoperative TXA dose was administered. Patients in the non-TXA group underwent the same type of debridement during the same period but did not receive perioperative TXA.

### Surgical procedure and postoperative management

All patients underwent debridement surgery. The procedures were performed by the same orthopaedic infection treatment team. The surgical approach was selected according to previous incisions, preoperative imaging assessment, and intraoperative exposure requirements. The infected area was fully exposed, and sinus tracts, scar tissue, infected granulation tissue, necrotic bone, devitalized soft tissue, and suspicious infected tissue were thoroughly removed. In patients with intramedullary involvement, canal opening, curettage, reaming, or other lesion-directed procedures were performed according to the extent of infection.

For patients who had been receiving systemic antimicrobial therapy before admission, antimicrobial treatment was discontinued on admission before intraoperative sampling. Because the interval from admission to surgery varied among patients, no fixed preoperative antibiotic-free interval was imposed. Multiple deep bone or soft-tissue specimens were routinely collected intraoperatively for bacterial culture and antimicrobial susceptibility testing, and histopathological examination was performed when necessary. After completion of debridement and specimen collection, the surgical field was repeatedly irrigated using normal saline (>3,000 mL), hydrogen peroxide, and povidone-iodine. The same irrigation approach was used in both groups. Local stabilization or reconstruction was selected according to the extent of bone defect, local stability, and soft-tissue conditions. When stabilization was required, plate fixation was used; patients not requiring additional stabilization were managed without fixation. Antibiotic-loaded polymethylmethacrylate (PMMA) bone cement was prepared using a size-40 package of PALACOS® R + G (Heraeus Medical GmbH, Wehrheim, Germany), a commercially premixed gentamicin-containing high-viscosity PMMA cement containing 0.5 g of gentamicin base, with an additional 5 g of vancomycin mixed into each package. The same dual-antibiotic PMMA formulation was used in both groups. A drainage tube was placed according to the wound condition, and the incision was closed in layers.

Postoperative systemic antimicrobial therapy was guided by intraoperative deep-tissue culture and antimicrobial susceptibility results. For culture-positive cases, systemic antimicrobial therapy was selected according to the identified pathogen and antimicrobial susceptibility profile; the agents used included vancomycin, cefazolin, linezolid, levofloxacin, and cefoperazone/sulbactam. For culture-negative cases, ceftriaxone was used empirically according to institutional practice. Systemic antimicrobial therapy was generally continued for approximately 6 weeks. Complete blood count, C-reactive protein, erythrocyte sedimentation rate, hemoglobin level, platelet count, prothrombin time, and fibrinogen level were regularly monitored during the perioperative period. Drainage tubes were generally retained for approximately 7 days and were removed according to the local wound and drainage conditions.

### Second-stage surgery and frozen-section assessment

All included patients were managed using a planned two-stage surgical strategy established at the time of first-stage debridement, and all subsequently underwent the planned second-stage procedure. The timing of the second-stage procedure was individualized and was determined after clinical assessment indicated adequate infection control, based on wound healing, local signs and symptoms, changes in inflammatory markers, imaging findings, and the surgeon’s overall judgment. No fixed interval between first-stage debridement and the planned second-stage procedure was mandated. During the planned second-stage procedure, the antibiotic-loaded PMMA cement was removed and the local bone and soft-tissue conditions were reassessed. Tissue specimens were routinely obtained from the bone ends and surrounding soft tissues for intraoperative frozen-section examination to assess residual acute inflammation. Frozen sections were evaluated by pathologists who were unaware of TXA exposure. Neutrophil counts were reported as the number of neutrophils per high-power field (HPF) at ×400 magnification. For each patient, the highest pathologist-reported neutrophil count per HPF among the examined frozen-section specimens was used as the recorded second-stage frozen-section neutrophil count.

### Outcome definitions

The primary outcomes comprised three infection-control-related outcomes—second-stage frozen-section neutrophil count, unplanned repeat debridement, and infection recurrence during the observation period—and venous thromboembolism as a thromboembolic safety outcome. Unplanned repeat debridement was defined as any additional debridement performed after first-stage debridement but before the planned second-stage procedure because of suspected persistent or recurrent infection, wound problems, purulent drainage, abnormal inflammatory markers, or imaging findings suggestive of uncontrolled infection. The planned second-stage procedure itself was not classified as unplanned repeat debridement.

For recurrence assessment, the observation period extended from first-stage debridement through the last valid follow-up. Recurrence was defined as the reappearance of clinical or investigative evidence of infection after a period of clinical infection control following first-stage debridement, including new or recurrent local redness, swelling, warmth, pain, sinus formation, purulent drainage, renewed elevation of inflammatory markers, imaging evidence of infection progression, or the need for additional surgical treatment, supported by microbiological, histopathological, or intraoperative evidence when available. The timing of each recurrence relative to the planned second-stage procedure was recorded. The last follow-up was defined as the latest outpatient visit, inpatient review, imaging assessment, or telephone follow-up after the planned second-stage procedure at which a definite infection-control status could be obtained. Post-second-stage follow-up duration was calculated from the planned second-stage procedure to the last valid follow-up and was at least 12 months for all patients. For patients with recurrence, the interval from first-stage debridement to the diagnosis of recurrence was recorded in weeks as the recurrence time. Because the number of recurrence events was small, Kaplan–Meier survival analysis was not performed. Instead, the occurrence of infection recurrence was compared as a binary outcome, and recurrence time was described.

Secondary outcomes included operative time, estimated intraoperative blood loss, postoperative drainage volume, recorded perioperative blood loss, and postoperative transfusion. Estimated intraoperative blood loss was obtained from the anesthesia record and was based on the volume of suctioned fluid after subtraction of the recorded irrigation volume, together with the estimated blood content of surgical gauze. Postoperative drainage volume was obtained from nursing records and was defined as the cumulative drainage volume recorded until drain removal. Recorded perioperative blood loss was defined as the sum of estimated intraoperative blood loss and cumulative postoperative drainage volume. Red-blood-cell transfusion was generally indicated when the postoperative hemoglobin level was <70 g/L, or when the hemoglobin level was 70–80 g/L in the presence of clinically relevant symptoms or signs of anemia. Venous thromboembolism was assessed from first-stage debridement through the last valid follow-up based on clinical symptoms and, when clinically indicated, vascular ultrasonography or other relevant imaging examinations. Routine screening imaging for asymptomatic venous thromboembolism was not performed.

### Statistical analysis

Statistical analyses were performed using the pandas and scipy packages in Python. Continuous variables were expressed as mean ± standard deviation or median [interquartile range] according to distribution characteristics. Continuous variables with approximately normal or relatively symmetric distributions were compared using Welch’s *t*-test. Non-normally distributed continuous variables were compared using the Mann–Whitney U test. Categorical variables were expressed as n/N (%) and compared using the chi-square test or Fisher’s exact test. Unadjusted effect estimates were expressed as TXA minus non-TXA. For continuous outcomes analyzed using Welch’s *t*-test, mean differences and corresponding 95% confidence intervals were calculated using Welch’s method. For second-stage frozen-section neutrophil count, the between-group effect estimate was expressed as the Hodges–Lehmann location shift, with the 95% confidence interval estimated using 10,000 bootstrap resamples. For binary outcomes, effect estimates were expressed as absolute risk differences with Newcombe 95% confidence intervals. When no events occurred in either group, no between-group hypothesis test was performed, and the absolute risk difference with its 95% confidence interval was reported descriptively. Because of the small numbers of infection recurrence and unplanned repeat debridement events and the absence of venous thromboembolic events, multivariable logistic regression was not performed for these binary outcomes to avoid model overfitting. All tests were two-sided, and *P* < 0.05 was considered statistically significant. All variables included in the analyses were complete for the final cohort of 82 patients; therefore, no missing-data imputation was performed. No *a priori* sample-size calculation was performed because of the retrospective cohort design. The study size was determined by all consecutive patients who met the predefined eligibility criteria during the study period.

The study was reported in accordance with the Strengthening the Reporting of Observational Studies in Epidemiology (STROBE) guideline, and the completed STROBE checklist is provided as supplementary material.

## Results

### Patient cohort and baseline characteristics

A total of 107 patients with humeral osteomyelitis were assessed for eligibility during the study period. Twenty-five patients were excluded: 3 because of distal humeral involvement, 6 because of acute hematogenous osteomyelitis, 7 because of incomplete clinical data, 6 because post-second-stage follow-up was less than 12 months after loss to follow-up and unsuccessful telephone contact, and 3 because of contraindications to TXA. The final cohort comprised 82 patients, including 47 in the TXA group and 35 in the non-TXA group. The overall median post-second-stage follow-up duration was 16.0 [15.0, 16.8] months. The median post-second-stage follow-up duration was 16.0 [15.0, 16.5] months in both the TXA and non-TXA groups, with no significant difference between groups (*P* = 0.785; Table 1).

**Table 1:** Baseline characteristics of the study cohort.

Variable	Total (*n* = 82)	TXA (*n* = 47)	Non-TXA (*n* = 35)	*P* value
Age, years	40.0 ± 8.6	40.0 ± 9.3	39.9 ± 7.7	0.994
Male sex, *n*/*N* (%)	49/82 (59.8)	28/47 (59.6)	21/35 (60.0)	0.969
Body mass index, kg/m²	24.0 ± 1.7	24.0 ± 1.8	23.9 ± 1.6	0.720
Smoking history, *n*/*N* (%)	35/82 (42.7)	21/47 (44.7)	14/35 (40.0)	0.672
Alcohol use, *n*/*N* (%)	20/82 (24.4)	12/47 (25.5)	8/35 (22.9)	0.780
Hypertension, *n*/*N* (%)	10/82 (12.2)	5/47 (10.6)	5/35 (14.3)	0.737
Diabetes mellitus, *n*/*N* (%)	4/82 (4.9)	1/47 (2.1)	3/35 (8.6)	0.308
Disease duration, months	15.0 [11.0, 25.2]	14.0 [11.0, 26.5]	15.0 [12.0, 24.5]	0.693
Etiology, *n*/*N* (%)				1.000
Post-traumatic osteomyelitis	75/82 (91.5)	43/47 (91.5)	32/35 (91.4)	
Chronic hematogenous osteomyelitis	7/82 (8.5)	4/47 (8.5)	3/35 (8.6)	
Previous operations, *n*	2.0 [1.0, 2.0]	2.0 [1.0, 2.0]	2.0 [1.0, 2.5]	0.696
Fixation method, *n*/*N* (%)				1.000
Plate fixation	77/82 (93.9)	44/47 (93.6)	33/35 (94.3)	
No fixation	5/82 (6.1)	3/47 (6.4)	2/35 (5.7)	
Preoperative hemoglobin, g/L	129.9 ± 9.0	129.8 ± 9.3	130.0 ± 8.7	0.944
Preoperative WBC, ×10^9^/L	7.5 [7.0, 8.2]	7.5 [7.0, 8.2]	7.6 [7.1, 8.1]	0.636
Preoperative CRP, mg/L	16.6 [12.1, 25.8]	14.5 [11.2, 24.6]	18.5 [13.3, 27.5]	0.117
Preoperative ESR, mm/h	29.0 [21.0, 38.0]	28.0 [20.0, 38.0]	29.0 [22.5, 40.0]	0.462
Preoperative platelet count, ×10^9^/L	255.8 ± 25.9	254.2 ± 26.4	257.9 ± 25.6	0.523
Preoperative prothrombin time, s	11.6 [11.4, 12.1]	11.9 [11.5, 12.4]	11.5 [11.3, 11.8]	<0.001
Preoperative fibrinogen, g/L	3.7 [3.4, 4.0]	3.6 [3.3, 4.0]	3.7 [3.4, 4.0]	0.421
Post-second-stage follow-up duration, months	16.0 [15.0, 16.8]	16.0 [15.0, 16.5]	16.0 [15.0, 16.5]	0.785

Values are presented as mean ± standard deviation, median [interquartile range], or *n*/*N* (%), as appropriate. *P* values were calculated using Welch’s *t*-test, Mann–Whitney *U*-test, *χ*² test, or Fisher’s exact test, as appropriate.

Measured baseline characteristics were generally similar between the two groups. The distributions of osteomyelitis etiology and fixation method were also similar between groups (both *P* = 1.000; Table 1). The mean age was 40.0 ± 9.3 years in the TXA group and 39.9 ± 7.7 years in the non-TXA group (*P* = 0.994). Male patients accounted for 28/47 (59.6%) in the TXA group and 21/35 (60.0%) in the non-TXA group (*P* = 0.969). No significant differences were observed between groups in body mass index, smoking history, alcohol use, hypertension, diabetes mellitus, disease duration, previous operations, preoperative hemoglobin level, white blood cell count, C-reactive protein, erythrocyte sedimentation rate, platelet count, or fibrinogen level (Table 1).

Preoperative prothrombin time differed significantly between the two groups. The preoperative prothrombin time was 11.9 [11.5, 12.4] s in the TXA group and 11.5 [11.3, 11.8] s in the non-TXA group (*P* < 0.001; Table 1). However, the values in both groups were within a clinically acceptable range, and patients with obvious coagulation abnormalities were not included in this study.

### Perioperative blood-loss outcomes

Representative intraoperative photographs from the TXA and non-TXA groups are shown in Figure 1 for visual context only; no standardized intraoperative visual-field clarity score was available. The TXA group showed lower perioperative blood-loss measures and shorter operative time than the non-TXA group. Operative time was significantly shorter in the TXA group than in the non-TXA group (139.2 ± 13.4 min vs. 148.7 ± 11.8 min, *P* = 0.001). Estimated intraoperative blood loss was significantly lower in the TXA group than in the non-TXA group (289 ± 35 mL vs. 346 ± 49 mL, *P* < 0.001; Table 2).

**Figure 1:**
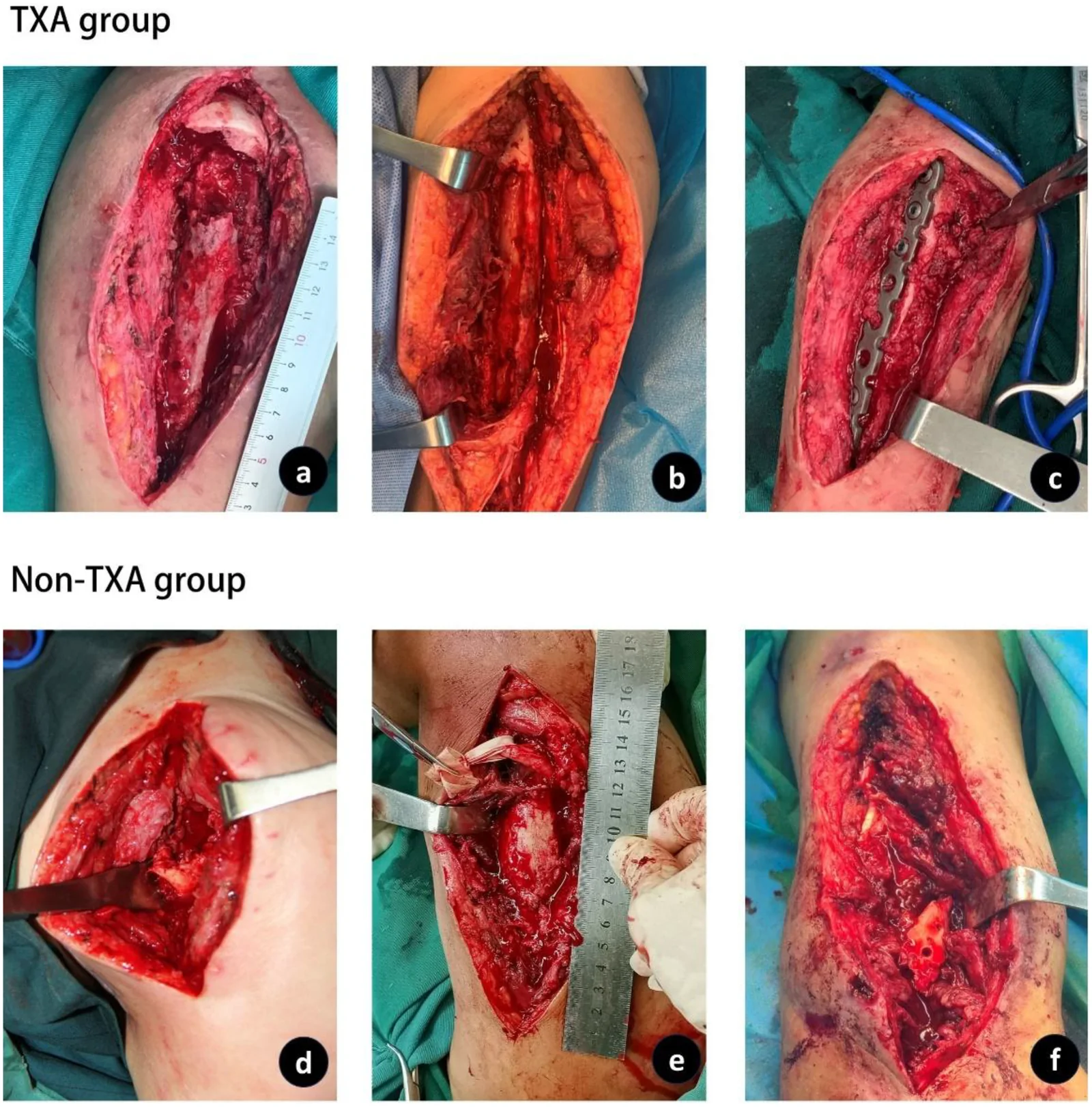
Representative intraoperative photographs from the TXA and non-TXA groups. **(a–c)** Representative surgical fields from patients who received preoperative intravenous tranexamic acid (TXA). **(d–f)** Representative surgical fields from patients who did not receive TXA. These images illustrate intraoperative surgical fields in the two groups and are provided for visual context only. Formal quantitative comparisons of perioperative blood-management outcomes are provided in Figure 2 and Table 2.

**Table 2:** Perioperative blood-management outcomes.

Variable	Total (*n* = 82)	TXA (*n* = 47)	Non-TXA (*n* = 35)	Effect estimate (95% CI)	*P* value
Operative time, min	143.3 ± 13.5	139.2 ± 13.4	148.7 ± 11.8	−9.5 (−15.1 to −4.0)	0.001
Estimated intraoperative blood loss, mL	313 ± 50	289 ± 35	346 ± 49	−57 (−76 to −37)	<0.001
Postoperative drainage volume, mL	168 ± 27	160 ± 26	179 ± 23	−19 (−30 to −8)	<0.001
Recorded perioperative blood loss, mL	481 ± 62	449 ± 47	525 ± 53	−76 (−98 to −53)	<0.001
Postoperative transfusion, *n*/*N* (%)	9/82 (11.0)	4/47 (8.5)	5/35 (14.3)	−5.8% (−21.7% to 8.2%)	0.486

Values are presented as mean ± standard deviation or *n*/*N* (%), as appropriate. Blood-volume measures and their effect estimates are reported to the nearest milliliter to reflect the precision of the underlying clinical measurements. Effect estimates are unadjusted and expressed as TXA minus non-TXA. For continuous outcomes, effect estimates are mean differences with 95% confidence intervals calculated using Welch’s method. For postoperative transfusion, the effect estimate is the absolute risk difference with a Newcombe 95% confidence interval. *P* values were calculated using Welch’s *t*-test or Fisher’s exact test, as appropriate. CI, confidence interval; TXA, tranexamic acid.

Postoperative drainage volume was also significantly lower in the TXA group than in the non-TXA group (160 ± 26 mL vs. 179 ± 23 mL, *P* < 0.001). Accordingly, recorded perioperative blood loss was significantly lower in the TXA group than in the non-TXA group (449 ± 47 mL vs. 525 ± 53 mL, *P* < 0.001). Postoperative transfusion was required in 4/47 patients (8.5%) in the TXA group and 5/35 patients (14.3%) in the non-TXA group, with no significant difference between groups (*P* = 0.486; Table 2).

Figure 2 shows the distribution of perioperative blood-management outcomes between the two groups. The box-and-scatter plots demonstrated lower overall distributions of operative time, estimated intraoperative blood loss, postoperative drainage volume, and recorded perioperative blood loss in the TXA group than in the non-TXA group (Figure 2).

**Figure 2:**
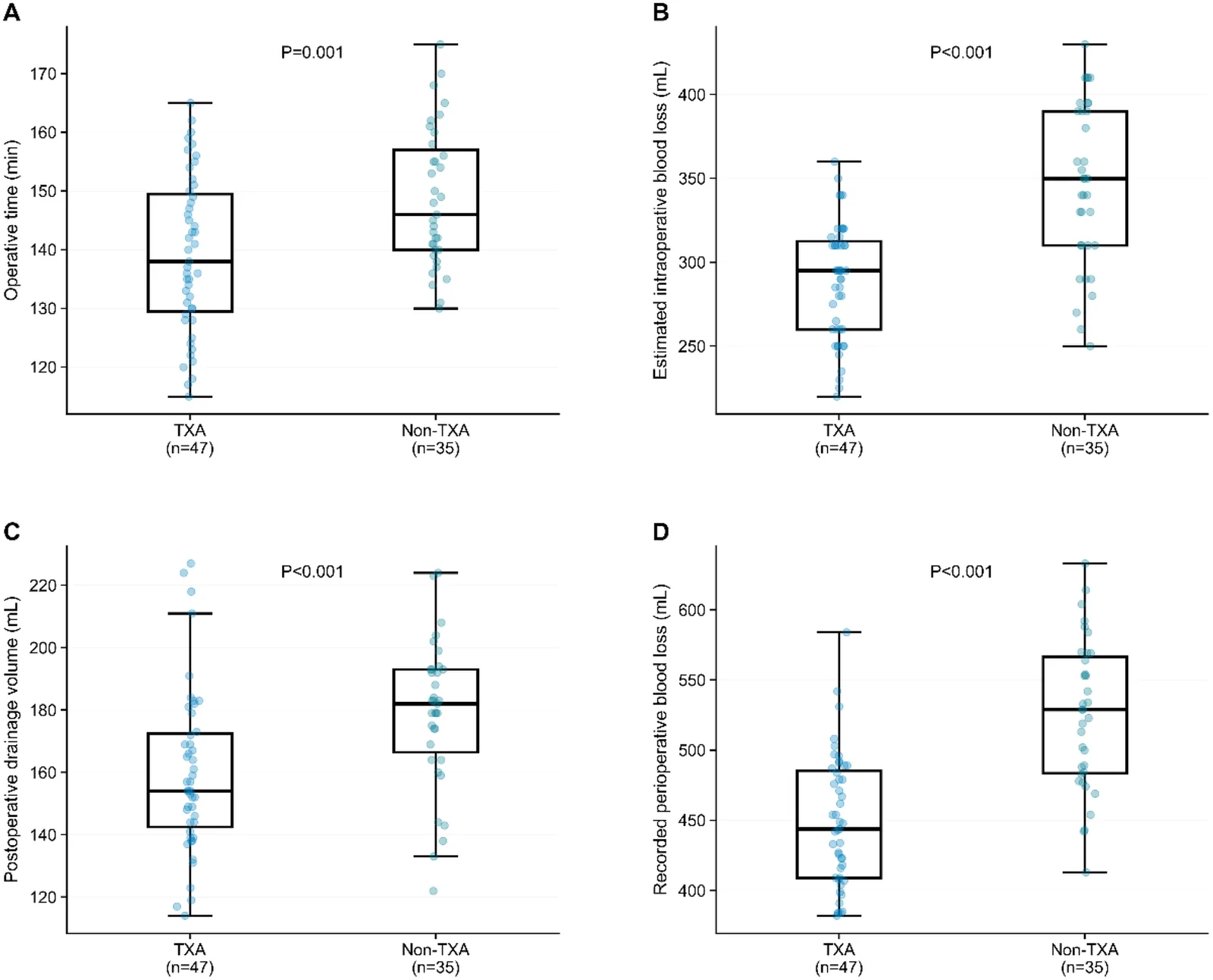
Perioperative blood-management outcomes. Box-and-scatter plots show operative time **(A)**, estimated intraoperative blood loss **(B)**, postoperative drainage volume **(C)**, and recorded perioperative blood loss **(D)**. Boxes represent the interquartile range, horizontal lines within boxes represent medians, and each dot represents one patient. *P* values were calculated using Welch’s *t*-test.

### Infection-control and safety outcomes

All 82 patients completed the planned second-stage procedure and underwent frozen-section assessment at that stage. Second-stage frozen-section neutrophil count did not differ significantly between the two groups. The frozen-section count was 2.0 [1.0, 2.0] in the TXA group and 2.0 [1.0, 3.0] in the non-TXA group (*P* = 0.106; Table 3).

**Table 3:** Infection-control and safety outcomes.

Variable	Total (*n* = 82)	TXA (*n* = 47)	Non-TXA (*n* = 35)	Effect estimate (95% CI)	*P* value
Second-stage frozen-section neutrophil count, neutrophils/HPF	2.0 [1.0, 3.0]	2.0 [1.0, 2.0]	2.0 [1.0, 3.0]	0.0 (−1.0 to 0.0)	0.106
Unplanned repeat debridement, n/N (%)	7/82 (8.5)	4/47 (8.5)	3/35 (8.6)	−0.1% (−14.8% to 12.7%)	1.000
Infection recurrence during the observation period, n/N (%)	8/82 (9.8)	4/47 (8.5)	4/35 (11.4)	−2.9% (−18.3% to 10.4%)	0.718
Venous thromboembolism, n/N (%)	0/82 (0.0)	0/47 (0.0)	0/35 (0.0)	0.0% (−9.9% to 7.6%)	—

Values are presented as median [interquartile range] or *n*/*N* (%), as appropriate. Effect estimates are unadjusted and expressed as TXA minus non-TXA. For the second-stage frozen-section neutrophil count, the effect estimate is the Hodges–Lehmann location shift; the 95% confidence interval was estimated by bootstrap resampling (10,000 resamples). For binary outcomes, effect estimates are absolute risk differences with Newcombe 95% confidence intervals. *P* values were calculated using the Mann–Whitney *U*-test or Fisher’s exact test, as appropriate. No between-group hypothesis test was performed for venous thromboembolism because no events occurred in either group. CI, confidence interval; TXA, tranexamic acid. All eight recurrence events occurred before the planned second-stage procedure; no recurrence was documented during at least 12 months of subsequent post-second-stage follow-up.

Before the planned second-stage procedure, unplanned repeat debridement was required in 4/47 patients (8.5%) in the TXA group and 3/35 patients (8.6%) in the non-TXA group, with no significant difference between groups (*P* = 1.000). Infection recurrence during the observation period occurred in 4/47 patients (8.5%) in the TXA group and 4/35 patients (11.4%) in the non-TXA group, with no statistically significant difference between groups (*P* = 0.718). All eight recurrence events occurred after a period of clinical infection control following first-stage debridement but before the planned second-stage procedure. Of these eight patients, seven underwent unplanned repeat debridement, whereas one did not undergo additional debridement. Among patients with recurrence, the time from first-stage debridement to recurrence ranged from 7 to 11 weeks in the TXA group and from 4 to 11 weeks in the non-TXA group. No infection recurrence was documented after the planned second-stage procedure during subsequent follow-up, which was at least 12 months for all patients (Table 3).

No clinically diagnosed venous thromboembolic events were documented in either group during follow-up [0/47 vs. 0/35; absolute risk difference, 0.0% (95% CI, −9.9% to 7.6%); Table 3]. These findings are summarized in Tables 2, 3.

## Discussion

This study focused on debridement for chronic osteomyelitis of the proximal and mid-humerus, a specific scenario in orthopaedic infection surgery. Unlike most previous studies that evaluated TXA primarily in terms of blood loss reduction and transfusion avoidance, the present study focused on its infection-control safety in an infected surgical field. Chronic osteomyelitis debridement is performed in a lesion environment with an established infectious burden, where necrotic bone, infected granulation tissue, fibrotic scar, potential biofilm, and residual bacteria may coexist (4, 19). Therefore, in this setting, the key question is not merely whether TXA can exert a conventional hemostatic effect, but whether its antifibrinolytic action and stabilization of blood clots may adversely affect infectious focus clearance, residual inflammation, the need for repeat debridement, or infection recurrence during the observation period. In this study, no significant differences were observed between the TXA and non-TXA groups in second-stage frozen-section neutrophil count, unplanned repeat debridement, or infection recurrence during the observation period. No clinically diagnosed venous thromboembolic events were documented in either group. These findings suggest that, in this selected cohort, single-dose preoperative intravenous TXA was not associated with an obvious increase in infection recurrence after debridement for humeral chronic osteomyelitis. In addition, TXA use was associated with lower recorded perioperative blood-loss measures and shorter operative time, suggesting potential perioperative blood-management value in this setting (11, 12). Evidence from soft-tissue surgery has also been heterogeneous, with systemic TXA associated with reduced postoperative drainage and blood-loss-related measures in some body-contouring procedures, whereas a recent randomized trial in elective abdominoplasty found no significant reduction in perioperative blood loss or drain output, and a meta-analysis of randomized liposuction studies reported outcome-dependent benefits (20–22). However, the absolute between-group differences were modest, including a 9.5-min reduction in operative time and an approximately 57-mL reduction in estimated intraoperative blood loss, and no statistically detectable difference in postoperative transfusion was observed. Accordingly, the clinical relevance of these perioperative differences should be interpreted cautiously rather than inferred solely from statistical significance.

Mechanistically, TXA competitively binds to the lysine-binding sites of plasminogen, thereby inhibiting the conversion of plasminogen to plasmin, reducing fibrin degradation, and stabilizing blood clots (8). This mechanism forms the basis for its effect in reducing perioperative bleeding and postoperative oozing. In the context of debridement in an infected field, TXA may theoretically have a dual influence on infection control. On the one hand, reducing diffuse bleeding and postoperative hematoma formation may decrease local blood accumulation, dead-space exudation, and persistent inflammatory stimulation. Local hematoma or blood accumulation may provide a permissive microenvironment for bacterial persistence, proliferation, and biofilm maintenance; therefore, reducing hematoma formation may be beneficial for wound-healing conditions (23–25). On the other hand, TXA acts by stabilizing fibrin and blood clots, which raises a theoretical concern in infected surgery: a stable fibrin–clot environment might influence residual bacterial clearance, local antibiotic penetration, or inflammatory-cell access to the infected area. Experimental evidence has shown that Staphylococcus aureus can persist within fibrin networks, supporting the biological plausibility of this concern, although direct clinical evidence in osteomyelitis debridement remains limited (26). Therefore, TXA use in chronic osteomyelitis debridement should not be regarded simply as an extension of its role in aseptic orthopaedic surgery, but should first be evaluated as a safety issue in an infected surgical environment.

By using second-stage frozen-section neutrophil count, unplanned repeat debridement, and infection recurrence as infection-control-related indicators, the present study attempted to answer whether TXA use was associated with unfavorable infection-control signals. Frozen-section histology and neutrophil counts are widely used as adjunctive indicators of residual infection or inflammation in bone and joint infection diagnosis, although their interpretation depends on sampling site, diagnostic threshold, and pathological consistency (27–29). Importantly, the present findings only indicate that no unfavorable infection-control signal associated with TXA was observed; they do not imply that TXA has an anti-infective effect or can reduce recurrence. The main determinants of infection control remain adequate debridement, effective dead-space management, stable reconstruction, appropriate antimicrobial therapy, and viable, well-vascularized soft-tissue coverage, rather than the hemostatic agent itself (3, 4).

Debridement for chronic osteomyelitis of the proximal and mid-humerus has bleeding-management characteristics that differ from those of lower-limb long bones and conventional elective procedures. This region is covered by abundant musculature and is adjacent to important neurovascular structures, making it difficult to obtain a stable bloodless field using a tourniquet. Infected granulation tissue, periosteal stripping, intramedullary canal exposure, and scar release can all contribute to diffuse bleeding. Persistent oozing not only increases recorded perioperative blood loss and postoperative drainage, but also increases suctioning, wiping, electrocoagulation, and ligation, which may interfere with the recognition of infected tissue boundaries and reduce the controllability of the debridement process. Therefore, the potential value of TXA in this setting may not be limited to reducing transfusion demand; it may also be reflected in reducing diffuse oozing, decreasing postoperative drainage, reducing repeated hemostatic maneuvers, and shortening operative time (9, 10, 20). However, because this was a retrospective study without a standardized intraoperative visual-field clarity score, the present data cannot directly prove that TXA improved the operative field. Estimated intraoperative blood loss, postoperative drainage volume, recorded perioperative blood loss, and operative time should be interpreted only as indirect indicators of the potential perioperative effect of TXA on the debridement process.

Venous thromboembolism is a safety issue that must be considered when TXA is used in orthopaedic surgery. Because TXA inhibits fibrinolysis, it may theoretically increase thrombotic risk, particularly in patients with a history of VTE, prolonged immobilization, lower-limb surgery, advanced age, cardiovascular or cerebrovascular disease, renal dysfunction, or long-term anticoagulant therapy (30–32). The present study deliberately focused on chronic osteomyelitis of the proximal and mid-humerus, which reduced the confounding effect of the naturally higher VTE risk associated with femoral, tibial, or other lower-limb procedures. If femoral or other lower-limb osteomyelitis cases had been included in the same analysis, anatomical site, postoperative weight-bearing restriction, and baseline lower-limb thrombosis risk could have become strong confounders and weakened the interpretability of TXA safety. Therefore, although the single-humerus scenario limited the sample size, it helped maintain a more focused clinical question and better matched the focus of this study: infection-control safety of TXA in non-tourniquet-dependent upper-limb debridement. No clinically diagnosed venous thromboembolic events were documented in either group in this cohort; nevertheless, given the limited sample size and low event rate, this finding should be interpreted as a preliminary safety signal rather than definitive exclusion of rare thrombotic risk.

This study has several limitations. First, this was a single-center retrospective cohort study, and TXA use was not randomly assigned. Although measured baseline characteristics were generally similar between groups, the specific rationale for TXA prescribing was not systematically documented for every patient; therefore, prescriber-related selection bias and other unmeasured confounding cannot be excluded. Second, the sample size was limited, and the numbers of infection-recurrence and unplanned repeat-debridement events were small; no clinically diagnosed VTE events were documented. These low event counts limited statistical precision and the ability to detect rare adverse events. In addition, because vascular imaging was performed only when clinically indicated rather than as routine screening, asymptomatic venous thromboembolic events may have been missed. Third, although second-stage frozen-section histology can serve as an auxiliary indicator of residual local inflammation, its interpretation may be affected by sampling location, diagnostic threshold, and interobserver consistency, and it cannot replace clinical recurrence assessment throughout the observation period (27, 28). Fourth, this study did not use a standardized intraoperative visual-field clarity score; therefore, the perioperative value of TXA could only be inferred indirectly from blood loss, drainage volume, and operative time. In addition, although estimated intraoperative blood loss was based on suctioned fluid volume after subtraction of irrigation volume together with the estimated blood content of surgical gauze, some measurement error was unavoidable.

## Conclusions

In patients without contraindications to tranexamic acid, a single preoperative intravenous dose of tranexamic acid was associated with lower estimated intraoperative blood loss, postoperative drainage volume, and recorded perioperative blood loss during debridement for proximal-to-mid humeral chronic osteomyelitis, as well as shorter operative time. In this selected cohort, no statistically detectable increase was observed in second-stage frozen-section neutrophil counts, unplanned repeat debridement, or infection recurrence during the observation period. No clinically diagnosed venous thromboembolic events were documented in either group. These findings suggest that tranexamic acid may represent a potential perioperative blood-management option for this type of infected debridement; however, its infection-control safety should be further validated in larger, multicenter prospective studies.

## Data Availability

The raw data supporting the conclusions of this article will be made available by the authors, without undue reservation.
